# The negative effects of COVID-19 and national lockdown on emergency surgery morbidity due to delayed access

**DOI:** 10.1186/s13017-021-00382-z

**Published:** 2021-07-13

**Authors:** Francesco A. Ciarleglio, Marta Rigoni, Liliana Mereu, Cai Tommaso, Alessandro Carrara, Gianni Malossini, Saverio Tateo, Giuseppe Tirone, Truls E. Bjerklund Johansen, Pier Paolo Benetollo, Antonio Ferro, Giovanni Maria Guarrera, Mario Grattarola, Giandomenico Nollo, Alberto Brolese

**Affiliations:** 1 General Surgery II & HPB Unit, Azienda Provinciale per i Servizi Sanitari (APSS), Santa Chiara Hospital, Largo Medaglie d’Oro, 1, 38122 Trento, Italy; 2 IRCS – Innovation and Clinical Health Research - Bruno Kessler Foundation (FBK), 38123 Trento, Italy; 3 Department of Industrial Engineering, BIOtech Lab, University of Trento, 38122 Trento, Italy; 4 Obstetrics and Gynaecology Unit, Azienda Provinciale per i Servizi Sanitari (APSS), Santa Chiara Hospital, 38122 Trento, Italy; 5 Urology Unit, APSS, Azienda Provinciale per i Servizi Sanitari (APSS), Santa Chiara Hospital, 38122 Trento, Italy; 6 General Surgery I & Thoracic Unit, Azienda Provinciale per i Servizi Sanitari (APSS), Santa Chiara Hospital, 38122 Trento, Italy; 7 Institute of Clinical Medicine, University of Oslo, Oslo, Norway; 8 Department of Urology, Oslo University Hospital, Oslo, Norway; 9 Institute of Clinical Medicine, University of Aarhus, Aarhus, Denmark; 10 Provincial Health Care Agency Staff Management, Azienda Provinciale per i Servizi Sanitari (APSS), Santa Chiara Hospital, Trento, Italy

**Keywords:** COVID-19 disease, Surgical care, Delayed access, Emergency surgery, Complications

## Abstract

**Background:**

The aim of this retrospective comparative study was to assess the impact of COVID-19 and delayed emergency department access on emergency surgery outcomes, by comparing the main clinical outcomes in the period March–May 2019 (group 1) with the same period during the national COVID-19 lockdown in Italy (March–May 2020, group 2).

**Methods:**

A comparison (groups 1 versus 2) and subgroup analysis were performed between patients’ demographic, medical history, surgical, clinical and management characteristics.

**Results:**

Two-hundred forty-six patients were included, 137 in group 1 and 109 in group 2 (p = 0.03). No significant differences were observed in the peri-operative characteristics of the two groups. A declared delay in access to hospital and preoperative SARS-CoV-2 infection rates were 15.5% and 5.8%, respectively in group 2. The overall morbidity (OR = 2.22, 95% CI 1.08–4.55, p = 0.03) and 30-day mortality (OR = 1.34, 95% CI 0.33–5.50, =0.68) were significantly higher in group 2. The delayed access cohort showed a close correlation with increased morbidity (OR = 3.19, 95% CI 0.89–11.44, p = 0.07), blood transfusion (OR = 5.13, 95% CI 1.05–25.15, p = 0.04) and 30-day mortality risk (OR = 8.00, 95% CI 1.01–63.23, p = 0.05). SARS-CoV-2-positive patients had higher risk of blood transfusion (20% vs 7.8%, p = 0.37) and ICU admissions (20% vs 2.6%, p = 0.17) and a longer median LOS (9 days vs 4 days, p = 0.11).

**Conclusions:**

This article provides enhanced understanding of the effects of the COVID-19 pandemic on patient access to emergency surgical care. Our findings suggest that COVID-19 changed the quality of surgical care with poorer prognosis and higher morbidity rates. Delayed emergency department access and a “filter effect” induced by a fear of COVID-19 infection in the population resulted in only the most severe cases reaching the emergency department in time.

## Background

SARS-CoV-2 virus infection and the related clinical signs of disease started in Wuhan, Hubei, China, in late 2019 and soon spread rapidly all over the world [1]. On March 11, 2020, the World Health Organization (WHO) declared the infection a pandemic disease [2]. COVID-19 became a public health crisis that has profoundly modified medical and surgical patient management. The Italian Government imposed a lockdown with significant restrictions on human contact, travel and business operations on March 10, 2020. Between March and May 2020, Italy was the third most affected country after the USA and Spain with 204,576 confirmed disease cases and 26,049 deaths [3]. The Italian Health Service’s response to COVID-19 and the related challenges have resulted in considerable changes in hospital activities. The vast number of COVID-19 patients requiring hospitalisation and critical care exceeded the capacity of Italian hospitals. The most significant consequence was the discontinuation of elective non-oncological surgical procedures and the switch from non-COVID-19 pathways to COVID-19 pathways in accordance with international guidelines for all patients admitted to hospital [4]. Emergency department (ED) and urgent surgical protocols were modified by the health services of all Italian regions resulting in a complete redistribution and reorganisation of activities and services. Our hospital is a regional hub for emergency surgery. During the first wave of the COVID-19 pandemic, all spoke hospitals were promptly transformed into COVID hospitals, meaning that a far higher number of emergency surgical procedures had to be performed at the regional hub centre, with potentially unfavourable and unpredictable effects on patient outcomes.

We hypothesised that the Italian government lockdown imposed during the initial stages of the COVID-19 crisis limited free access to the hospital by emergency surgery patients, which in turn resulted in increased intra-operative complications, overall morbidity, increased length of hospital stay, ICU stay and mortality. The aim of this retrospective comparative study was to assess how the COVID-19 pandemic and related containment measures imposed in Italy affected emergency surgery outcomes also through limited or delayed ED access in our tertiary teaching regional hub hospital, by comparing data on the clinical and management outcomes of the Italian COVID-19 lockdown period (March–May 2020) with the same period (March–May) of the previous year (2019).

## Materials and methods

### Patient setting and study schedule

This study was designed as a retrospective cohort study comparing two separate groups of consecutive patients undergoing emergency surgery in a tertiary teaching regional hub hospital before (March–May 2019, group 1) and during the COVID-19 pandemic (March–May 2020, group 2). Patients > 18 years of age admitted to the ED of the regional hub hospital for emergency operations in the general, gynaecological or urological surgery departments were enrolled retrospectively. Exclusion criteria were age < 18 years, incomplete follow-up data, re-operations after elective surgery, outpatient clinic visits in the previous 7 days, patients on waiting list and day hospital admissions. Emergency surgery was defined as any emergency or urgent procedure performed by surgeons in an operating theatre under general anaesthesia. All emergency or urgent indications were eligible for inclusion, which was performed in accordance with the STROBE statement [5].

### Variables and outcomes

Patients’ demographic, medical history, surgical, clinical and management characteristics were recorded. The following scales, scores and parameters were recorded at the time of admission to the ED: Numeric Rating Scale (NRS) for pain [6], National Early Warning Score (NEWS) [7], Charlson Comorbidity index score (CCI) [8], declared delayed access > 48 h, patient diagnosis, presence/absence of sepsis criteria at admission [9], American Society of Anesthesiologists (ASA) score and SARS-CoV-2 swab test (for group 2 only). The surgical approach was classified as open, laparoscopic or endoscopic (operative cystoscopy, hysteroscopy and vaginal approach). The main outcomes were intraoperative complications, intensive care unit (ICU) admission, length of hospital stay (LOS), post-operative complications according to the Clavien-Dindo morbidity classification [10], intra/post-operative blood transfusion rate, in-hospital mortality and 30-day mortality. In-hospital mortality was defined as death during the same hospital stay and 30-day mortality was defined as death within 30 days after surgery. Thirty-day mortality rate was related to subjects affected by COVID-19 disease who underwent to emergency surgery procedures.

Clinical data were collected by reviewing all electronic medical records and, where necessary, by reviewing medical ward charts and/or by calling the patients.

Data collection was performed by the authors FAC, LM, AC and TC, and statistical analysis was performed by MR.

The study was approved by the Trento (Italy) Provincial Health Care Agency and Santa Chiara Hospital Ethical Committee (Declaration No. 5/2020) and data acquisition and storage were performed in compliance with Ethics Committee guidelines. The study was conducted in line with the Good Clinical Practice guidelines and the ethical principles enshrined in the Declaration of Helsinki. All medical history, clinical or laboratory data containing sensitive patient information were anonymised in order to ensure analysis of anonymous data only.

### Statistical analyses

The descriptive variables were expressed by mean ± standard deviation (SD) in the case of normal distribution or by median and first and third quartiles (q1, q3, respectively) in the case of non-normal distribution. The normality of the variables was tested with the Shapiro-Wilk test. The dichotomous variables or scores were expressed as frequency and occurrence rates. The characteristics of the patient populations were compared using the most appropriate test, depending on the nature and normality of the data (chi-squared test, Fisher exact test, Student t-test or U-Mann-Whitney test when appropriate).

Univariate and multivariate logistic regression models were used to evaluate the actual impact of the COVID-19 emergency on patient outcomes. When appropriate, multivariate models were used adjusting for ASA score, age, CCI at admission and NEWS at admission. When logistic regression was not feasible, we reported numbers, percentages, the p-value from Fisher exact test or median (q1-q3) and p-value from the U-Mann-Whitney test.

Two subgroup analyses were performed for group 2 (2020 patients). The first subgroup analysis evaluated the outcomes in relation to patients’ declared delayed admission, whereas the second was performed to assess the impact of SARS-CoV-2 positivity on patient outcomes versus the outcomes for SARS-CoV-2-negative patients.

A p-value of < 0.05 was set for statistical significance. Statistical analyses were performed using Stata software (StataCorp, 4905 Lakeway Drive, College Station, TX 77845, USA).

## Results

A total of 246 patients receiving emergency/urgent surgery were included, with 137 operations performed in the period March–May 2019 (group 1) and 109 in the period March–May 2020 (group 2) (COVID-19 pandemic).

### Patient characteristics, diagnoses and clinical management

No significant differences in the peri-operative characteristics were observed in the two groups in terms of age, gender, NRS, NEWS, CCI, ASA score, presence/absence of sepsis criteria, surgical approach or ICU admission. Declared delayed access to hospital was recorded in 0% of patients in group 1 and in 15.5% in group 2 (16 subjects, p < 0.01). All group 2 patients came from our region; therefore, the delays could not have resulted from patients coming from out of the area. Delayed access was reported by patients at ED admission and all data were recovered from ED admission charts. Indeed, every patient admitted during the COVID period was questioned regarding the presence of COVID-19 symptoms during triage screening and this procedure was duly recorded in the patients’ medical records. The reasons reported were delay in seeking medical assistance in 6 cases, avoided visiting the hospital for fear of COVID-19 infection in 5 cases and home management by the patient’s general practitioner in 5 cases.

SARS-CoV-2 infection (tested only in group 2) was diagnosed by preoperative nasopharyngeal swab in 5 out of 86 (5.8%) patients, with a statistically significant difference compared to the overall population (group 1, n = 137; group 2, n = 109; p = 0.03) (Tables 1 and 2).

**Table 1:** Type of diagnosis at admission

Diagnosis	Group 1 2019 n (%)	Group 2 2020 n (%)	p-value
**Overall population**	**137**	**109**	**0.03**
Trauma	4 (2.9)	4 (3.7)	0.73
Gastrointestinal perforation	8 (5.8)	5 (4.6)	0.78
Bowel obstruction	12 (8.8)	20 (18.3)	**0.03**
Hernia	5 (3.6)	4 (3.7)	1.00
Acute appendicitis	16 (11.7)	21 (19.3)	0.11
Acute cholecystitis	8 (5.8)	2 (1.8)	0.19
Bowel ischaemia	1 (0.7)	0 (0.0)	1.00
Testicular torsion	2 (1.5)	0 (0.0)	0.50
Urinary tract bleeding	9 (6.6)	2 (1.8)	0.12
Urinary tract obstruction	39 (28.5)	23 (21.1)	0.24
Extrauterine pregnancy	3 (2.2)	7 (6.4)	0.11
Pelvic inflammatory disease (PID)	3 (2.2)	5 (4.6)	0.47
Genital bleeding	2 (1.5)	2 (1.8)	1.00
Miscarriage	4 (2.9)	6 (5.5)	0.34
Ovarian benign disease	2 (1.5)	3 (2.7)	0.66
Other relevant surgical pathology	19 (13.9)	5 (4.6)	**0.02**

**Table 2:** Patient characteristics at admission

Characteristics	Group 1 2019 (137 Pts) n (%)	Group 2 2020 (109 Pts) n (%)	p-value
Age years, median (q1–q3)	53 (33–70)	48 (32–70)	0.41
Gender, n (%)			
Female	42 (30.7)	40 (36.7)	0.32
Male	95 (69.3)	69 (63.3)	
NRS median (q1–q3)			
≤3, n (%)	50 (37.3)	40 (36.7)	0.98
4–6, n (%)	34 (25.4)	29 (26.6)	
≥7, n (%)	50 (37.3)	40 (36.7)	
NEWS at admission, median (q1-q3)			
0–4, n (%)	101 (80.8)	86 (78.90)	0.39
5–6, n (%)	10 (8.0)	14 (12.8)	
≥7, n (%)	14 (11.2)	9 (8.3)	
CCI, median (q1-q3)	2 (0–5)	1 (0–5)	0.99
Declared delayed access > 48 h, n (%)	0 (0.0)	16 (15.5)	**< 0.01**
Sepsis criteria, n (%)	42 (31.3)	31 (28.7)	0.66
ASA score, median (q1-q3)	2 (1–3)	2 (1–3)	0.35
COVID-19 molecular swab test results, n (%)			
Negative	n.a.	81 (74.3)	
Positive	n.a.	5 (4.6)	
Not available	n.a.	23 (21.1)	

*q1* first quartile, *q3* third quartile, *SD* standard deviation

As regards diagnosis, differences in the percentages for group 1 and group 2 were observed for bowel obstruction (8.8% vs 18.3%), acute appendicitis (11.7% vs 19.4%), acute cholecystitis (5.8% vs 1.8%), urinary tract obstruction (28.5% vs 21.1%), extra-uterine pregnancy (2.2% vs 6.4%) and pelvic inflammatory disease (PID) (2.2% vs 4.6%) (Table 1). The surgical approaches used (percentages for group 1 vs group 2) were laparoscopic (35.8 vs 33.9%), open (16.8 vs 26.6%), laparoscopic and endoscopic procedures (35.8 vs 23.8%) and vaginal (3.6 vs 4.6%).

### Clinical outcomes

No differences between the two groups were observed in terms of intra-operative complications and LOS. In group 2 vs group 1, overall morbidity was significantly higher (more than double, OR = 2.22, 95% CI 1.08–4.55, p = 0.03), the risk of blood transfusion was 41% lower (OR = 0.59, 95% CI 0.24–1.43, p = 0.24) and 30-day mortality was 34% higher (OR = 1.34, 95%CI = 0.33–5.50, p = 0.68), although the intergroup differences for the two last variables were not statistically significant. The adjusted analysis for ASA score, age, CCI and NEWS confirmed the outcomes observed in the first crude analysis. Postoperative morbidity risk was higher in group 2 than in group 1 (aOR = 2.28, 95% CI 1.04–5.03, p = 0.04), but the blood transfusion rate and 30-day mortality risk were similar for the two groups. Group 2 post-operative morbidity and overall perioperative outcomes are shown in Tables 3 and 4, respectively.

**Table 3:** Group 2 post-operative morbidity

Description	Rate n, %
Overall	22/109, 20.2%
Post-operative bleeding	6/22, 27.4%
Pleural effusion	3/22, 13.6%
Pneumonia	3/22, 13.6%
Heart failure	2/22, 9.1%
Multiorgan failure due to septic shock	2/22, 9.1%
Acute peritonitis secondary to bowel perforation	2/22, 9.1%
Abdominal fascial dehiscence	2/22, 9.1%
Anastomotic leak	1/22, 4.5%
Kidney failure	1/22, 4.5%

**Table 4:** Comparison of clinical outcome: numbers, percentages, crude and adjusted outcome measurements for the two patient groups

Outcome	Group 1 2019 (137 Pts) n (%)	Group 2 2020 (109 Pts) n (%)	Crude OR (95% CI), p-value	Adjusted OR* (95% CI), p-value
ICU admissions, n (%)	9 (6.9)	3 (3.1)	N/A, p = 0.24	
Intra-operative complications, n (%)	0 (0)	1 (1)	N/A, p = 0.43	
Post-operative LOS, median (q1–q3)	3 (1-8)	4 (2-9)	N/A, p = 0.13	
Morbidity	15 (11.5)	22 (22.4)	OR = 2.22 (1.08–4.55), p = 0.03	aOR = 2.28 (1.04–5.03), p = 0.04
Dindo-Clavien 0–2	121 (93.1)	87 (88.8)		
Dindo-Clavien 3–4	8 (6.1)	9 (9.2)		
Dindo-Clavien 5	1 (0.8)	2 (2.0)		
Blood transfusion	17 (13.1)	8 (8.2)	OR = 0.59 (0.24–1.43), p = 0.24	aOR = 0.52 (0.19–1.40), p = 0.20
Death/in-hospital mortality	1 (0.7)	2 (1.8)	OR = 2.52 (0.23–28.20), p = 0.45	aOR = 4.38 (0.19–99.78), p = 0.35
30-day mortality	4 (3.1)	4 (4.1)	OR = 1.34 (0.33–5.50), p = 0.68	aOR = 1.65 (0.33–8.29), p = 0.54

*N/A* not available, *aOR* adjusted odds ratio, *CI* confidence interval, *OR* odds ratio

*Adjusted for ASA score, age, CCI and NEWS (216 patients)

### Subgroup analyses for group 2 subjects

Sixteen subjects in group 2 (15.5%) declared delayed access to the ED. According to ED admission time from onset symptoms, the declared delayed access group included 12 subjects who came to the ED after 72 to 96 h, 3 cases who came in after 96–120 h and one patient who came to the ED after more than 120 h. The most common diagnoses at admission were bowel obstruction (3 pts), gastrointestinal perforation (2 pts), complicated acute appendicitis (2 pts), acute cholecystitis (1 patient), incarcerated inguinal hernia (1 patient), extrauterine pregnancy (2 pts), PID (2 pts), genital bleeding (1 patient) and other surgical conditions (2 cases). In group 2, five subjects in the delayed access group declared they initially received home management from their GP. The diagnosis at admission to the ED was bowel obstruction in two cases, acute appendicitis with local peritonitis, PID and urinary tract obstruction in one case. The morbidity rate in the delayed access group was 41.7%. Two multi-organ failures secondary to septic shock, two basal pneumonia with respiratory failure and one kidney failure were recorded. A crude analysis of the delayed access cohort showed a three times higher risk of post-operative complications (OR = 3.19, 95% CI 0.89–11.44, p = 0.07). The blood transfusion risk was five times higher (OR = 5.13, 95% CI 1.05–25.15, p = 0.04), the 30-day mortality risk increased by eight times (OR = 8.00, 95%CI = 1.01–63.23, p = 0.05) and stoma risk for surgery unit patients was 59% higher (OR = 1.59, 95% CI 0.15–17.1, p = 0.70) for the delayed access group vs non-delayed-access complaints (Table 5). The adjusted analyses, however, reduced the entity of the differences and eliminated statistical significance (Table 5).

**Table 5:** Clinical outcomes analysis of group 2

	No declared delayed access n = 87 (84.5%)	Declared delayed access n = 16 (15.5%)	p-value	Crude OR (95% CI), p-value	Adjusted OR* (95% CI), p-value
Intra-operative complications, n (%)	0 (0.0)	1 (8.3)	0.13		
Morbidity, n (%)	15 (18.3)	5 (41.7)		OR = 3.19 (0.89–11.44) p = 0.07	aOR = 2.31 (0.51–10.45) p = 0.28
Dindo-Clavien 0–2, n (%)	73 (89.0)	10 (83.3)	**0.01**	n.a.	n.a.
Dindo-Clavien 3–4, n (%)	9 (11.0)	0 (0.0)			
Dindo-Clavien 5, n (%)	0 (0.0)	2 (16.7)			
Blood transfusions, n (%)	5 (6.10)	3 (25.0)		OR = 5.13 (1.05–25.15) **p = 0.04**	aOR = 3.70 (0.63–21.60) p = 0.15
30-day mortality, n (%)	2 (2.4)	2 (16.7)		OR = 8.00 (1.01–63.23) **p = 0.05**	aOR = 3.65 (0.24–54.69) p = 0.35
Stoma**	3 (6.5)	1 (10.0)		1.59 (0.15–17.1) p = 0.70	aOR = 1.11 (0.07–18.52) p = 0.94

*aOR* adjusted odds ratio, *CI* confidence interval, *OR* odds ratio

*Adjusted for ASA score, age, CCI and NEWS

**Only for operations performed in General Surgery Units

SARS-CoV-2-positive patients had higher risk for blood transfusion (20% vs 7.8%, p = 0.37) and ICU admission (20% vs 2.6%, p = 0.17) and a higher median LOS (9 days vs 4 days, p = 0.11), although these differences did not reach statistical significance. Table 6 shows the impact of SARS-CoV-2 positivity on sub-group 2 (SARS-CoV-2-negative versus SARS-CoV-2-positive).

**Table 6:** Impact of SARS-Cov-2 positivity on outcomes on group 2

Outcome	SARS-Cov-2 negative N = 81 (94.2)	SARS-Cov-2 positive N = 5 (5.8)	p-value	Crude OR (95% CI), p-value
Intra-operative complications, n (%)	0 (0.0)	1 (20.0)	0.06	n.a.
Post-operative morbidity, n (%)	19 (24.7)	0 (0.0)	0.58	n.a.
Dindo-Clavien 0–2, n (%)	67 (87.0)	5 (100.0)	1.0	n.a.
Dindo-Clavien 3–4, n (%)	8 (10.4)	0 (0.0)		
Dindo-Clavien 5, n (%)	2 (2.6)	0 (0.0)		
Blood transfusions, n (%)	6 (7.8)	1 (20.0)	0.37	2.96 (0.28–30.85), p = 0.37
30-day mortality, n (%)	3 (3.9)	0 (0.0)	1.0	n.a.
ICU admissions, n (%)	2 (2.6)	1 (20.0)	0.17	9.37 (0.69–126.56), p = 0.09
Declared Delayed access > 48 h, n (%)	9 (11.8)	1 (20.0)	0.49	1.29 (0.13–12.48), p = 0.82
Post-operative LOS, median (q1–q3)	4 (2–9)	9 (8-10)	0.11	n.a.

*CI* confidence interval, *ICU* intensive care unit, *LOS* length of stay, *OR* odds ratio, *q1* first quartile, *q3* third quartile

## Discussion

COVID-19 is a global pandemic disease that has brought major changes in human health services all around the world. In Italy, the exponential increase in severe cases led to a rise in hospitalisations with a progressive overcrowding in ICUs. As a consequence, we witnessed a switch from permanent emergency room ventilator stations to beds equipped with mechanical ventilation. This reorganisation resulted in an increased demand for dedicated ICU personnel and a rapid decrease in planned surgeries.

During the lockdown period, EDs experienced a large influx of patients with respiratory failure due to COVID-19-related pneumonia and a rapid increase in cases requiring immediate treatment in line with the growing outbreak. All medical services were promptly reorganised, especially those managing patients in need of surgical care [11]. Elective non-oncological surgery was temporarily put on stand-by, mainly in order to relocate staff, particularly anaesthesiologists, to help with emergency cases and to transform operating theatres into emergency rooms for the most severe COVID-19 patients. Non-operative management (NOM) of surgical patients had to be considered whenever possible. Only emergency cases and selected oncological procedures continued to take place.

In our region, the public health service closed five spoke EDs to concentrate emergency surgical activities in our hub hospital alone. Dedicated COVID-19 protocols was established. All surgical patients were screened for COVID-19 before admission to the ED. In order to prevent contamination in holding areas, no patients were transferred between different areas of the hospital until their destination had been confirmed as ready and a dedicated COVID-19 operating theatre was designated.

To our knowledge, this is one of the first studies analysing the impact of COVID-19 and the resulting lockdown on emergency surgical procedures in a single hub hospital by comparing two cohorts of patients enrolled in the same time periods before and during the pandemic (2019 versus 2020). A unique feature of our study is the comparison of outcomes in the same clinical units manned by the same senior surgical staff before and during the pandemic. Our findings demonstrate the consequences of reduced ED resources for ordinary non-COVID-19 patients in need of emergency surgery during the pandemic.

We observed an overall decrease in emergency surgical activities of 20.4% (p = 0.03) during the pandemic, which is comparable to other international findings [12, 13]. Nevertheless, an increase was observed in bowel obstruction, acute appendicitis, extra-uterine pregnancy and pelvic inflammatory disease (PID). Other authors have also observed a change in the clinical presentation of emergency cases during the COVID-19 pandemic, with a reduction in less severe conditions such as urinary tract conditions and an increase in certain surgical conditions, such as bowel obstruction, acute appendicitis, extra-uterine pregnancy and PID [14]. These observations most likely have a multifactorial explanation. The Italian Government issued declarations to the effect that the most effective way to save lives was for people to stay at home as far as possible. This restriction might explain the reduction in patients going to hospital for “non-essential” surgical consultations and trauma. Many people may have avoided going to hospital for fear of COVID-19 infection. General practitioners probably managed abdominal pain, pelvic pain and urinary burning symptoms at patients’ homes to a greater extent than before the pandemic. In group 2, 23 (21.1%) subjects declared first-line general practitioner management, but only 5 of these patient declared delayed access to the ED. Lifestyle changes during lockdown may explain the lower incidence of certain diseases such as acute cholecystitis. The lower rate of cholecystectomy during the pandemic might be due to NOM using percutaneous cholecystostomy in more cases. Another explanation could be that surgeons adopted a more conservative approach motivated by the fear of becoming patients themselves [15]. Estimates show that 85% of healthcare workers are exposed to the virus and the International Council of Nurses estimated an infection rate of 9% in Italy during the period March-April 2020 [16]. Lastly, as many elective procedures were postponed, fewer patients required emergency surgical revision due to complications. Interestingly, patients undergoing surgery were similar before and during the pandemic in terms of their gender, age, frailty and comorbidity, as assessed by NEWS and CCI [17]. The Italian World Bank Staff estimates that Italy has the second largest proportion of elderly adults in the world [18]. Consequently, Italian EDs are used to manage older subjects who are more susceptible to and more strongly affected by COVID-19, with at greater risk of developing emergency surgical conditions and related complications. In this study, patients’ median age was only slightly lower during the pandemic; however, no statistically significant differences were observed in terms of age.

A declared delayed access to ED of 48–92 h from the onset of clinical signs was observed in 15.5% of patients during the pandemic. Several factors could explain this finding, such as changes in outpatient pathways [19] and patients’ fear of going to hospitals and becoming infected with COVID-19 [20]. Many surgical patients were initially treated without surgery. Acute appendicitis, acute cholecystitis, uncomplicated diverticulitis and urinary tract infections treated using antibiotics alone. Consequently, many subjects were not referred to hospital until after NOM failure and in a worse clinical condition. Cano-Valderrama et al. [21] observed a 65.4% decrease in emergency surgical activity in a Spanish hospital due to delayed access. They also observed a higher proportion of emergency surgery cases without alternative treatments, such as intestinal obstruction and incarcerated hernia. Patients requiring surgical care therefore presented with more advanced disease due to delayed admission. This explains the poorer patient outcomes in the declared delayed access cohort in group 2 of our study. On the logistic regression analysis, delayed access was consistent with an increase in postoperative morbidity, blood transfusion and 30-day mortality rate. Open surgical approaches were used more frequently during the pandemic. This was necessary due to the clinical status of patients; however, we did not observe any significant difference in terms of mortality or morbidity compared with the data for 2019. However, patients in the open-surgery group had an increased crude blood transfusion rate, most likely due to a higher number of patients presenting with active bleeding, obstruction, perforation or requiring rapid damage control. Institutions like the Centers for Disease Control and Prevention in the USA and the American College of Surgeons [22] recommend using negative-pressure operating theatres for patients who are positive for or suspected of having COVID-19 infection. Authors of research papers [23] support this recommendation. The rationale is that pneumoperitoneum leakage may cause aerosol exposure for the surgical team during standard laparoscopy [24]. Active replication of the SARS-CoV-2 virus occurs in the respiratory and gastrointestinal tracts [25] and De Simone et al. [26] suggested that laparoscopy on COVID-19 patients should preferably be avoided, especially in emergency settings. The United Kingdom Royal College of Surgeons suggested considering minimally invasive surgery in highly selected individual cases, only where the clinical benefits for patients substantially exceed the risk of potential viral transmission [27]. Zheng et al. advised that caution should be exercised when performing laparoscopies, by limiting intra-abdominal pressure, reducing the electrocautery settings and minimising use of the Trendelenburg position [28]. A switch in surgical approach from minimally invasive to open was observed especially for procedures in general surgery or in the case of emergency bowel disease, in order to avoid intestinal spreading of the virus. However, to date, there is little evidence to support an increased risk of contamination amongst healthcare providers during laparoscopy, or of operating room pressure, surgical smoke, tissue extraction or CO_2_ deflation [29]. Therefore, abandoning laparoscopic surgery in favour of open surgery for fear of COVID-19 infection among staff is actually not justified. While median LOS was not statistically different between the two groups, a detailed analysis showed an increase in median LOS during the pandemic. Delayed access and increased morbidity justified a longer stay.

The morbidity rate increased during the pandemic, especially Dindo-Clavien score > 3. Subjects with older age, frailty, sepsis, delayed hospital access, comorbidities and those who were admitted during lockdown were at higher risk of complications. According to McLean et al. [14], patients requiring emergency surgery should report to hospital promptly and receive the surgical care that they require immediately. The risk of perioperative complications associated with COVID-19 disease is significant and can affect morbidity and mortality [30]. The COVID-19 Surg Collaborative group performed an international multicentre cohort study on patients undergoing surgery. In adjusted analyses of the COVID-19-positive test group, 30-day mortality and morbidity (Dindo-Clavien > 3) were associated with age > 70 years, ASA score > 3 and emergency surgery versus elective surgery (OR 1.67 [1.06–2.63], p = 0·026) [31]. The low number of COVID-19 patients in our series does not allow any conclusions in this respect. Indeed, the causes that led to complications not impacting on the death rate may be both clinical and statistical. Although complications were higher in group 2, since they occurred in hospitalised patients in a critical area, they were able to be diagnosed and therefore treated, thus limiting both in-hospital and 30-day mortality. The difference between the death rates of the two groups considered is not statistically different. This may be due to the small number of patients and low number of events recorded in the periods of time analysed.

## Study limitations

The retrospective and observational nature of our study could affect the general strength of the results. Due to the retrospective design, potentially useful data may have escaped collection, which may have limited in-depth analysis. On the other hand, the historical period of the first wave of the COVID-19 pandemic has to be analysed on the basis of real-world data.

Moreover, for the subgroup analyses, the small sample size and limited number of events did not allow us to draw solid conclusions. Some diagnoses and baseline characteristics were not homogeneous between groups, which limits the robustness of the results. However, a detailed description of the observed differences still provides clinically relevant data. This study reported results based on small numbers in a single centre. We believe that a national multicentre study with pooled data from a high number of institutions could be interesting and could provide more statistically reliable and robust results. Indeed, the COVID-19 pandemic is a global health emergency that generates a cascade of important health problems and deserves further analysis and comments.

## Conclusions

Our tertiary regional teaching hub hospital experienced a decrease in emergency surgical admissions and operations during the COVID-19 pandemic and lockdown period in the spring of 2020. SARS-CoV-2 infection was diagnosed preoperatively in 5/86 of operated patients (5.8%). Compared with the same period in 2019, there was an increase in the use of ambulances for pre-hospital patient transfer. A declared delay in access to hospital was recorded significantly more often during the pandemic (15.5%, p < 0.01). Our findings show that COVID-19 affected surgical care with higher morbidity rates due to more low-grade complications. Overall morbidity and 30-day mortality increased significantly. Delayed access correlated closely with increased morbidity, blood transfusion and 30-day mortality risk. SARS-CoV-2-positive patients had a higher risk for blood transfusions and ICU admission and a longer median LOS.

We believe delayed ED access is due to a “filter effect” motivated by the fear COVID-19 infection in the population. As a result, only the most severe cases were referred to the emergency department. Further research is needed to corroborate our assumption that the COVID-19 pandemic affected emergency surgery management and its outcomes. Further analysis of pooled data could allow the international community to better identify patients who should be referred promptly to emergency departments and which patients that may benefit from non-operative management.

## Data Availability

The datasets generated during and/or analysed during the current study are available from the corresponding author on reasonable request.

## Abbreviations

WHO: World Health Organization
ED: Emergency department
NRS: Numeric Rating Scale
NEWS: National Early Warning Score
CCI: Charlson Comorbidity Index score
ASA: American Society of Anesthesiologists
ICU: Intensive care unit
DRG: Diagnosis Related Groups
LOS: Length of hospital stay
NOM: Non-operative management
PID: Pelvic inflammatory disease
